# Construction and validation of a novel nomogram to predict cancer-specific survival in patients with gastric adenocarcinoma

**DOI:** 10.3389/fonc.2023.1114847

**Published:** 2023-02-09

**Authors:** Guole Nie, Honglong Zhang, Jun Yan, Danna Xie, Haijun Zhang, Xun Li

**Affiliations:** ^1^ The First School of Clinical Medicine, Lanzhou University, Lanzhou, China; ^2^ Department of General Surgery, The First Hospital of Lanzhou University, Lanzhou, China; ^3^ Key Laboratory of Biotherapy and Regenerative Medicine of Gansu Province, The First Hospital of Lanzhou University, Lanzhou, China

**Keywords:** gastric adenocarcinoma (GAC), cancer-specific survival (CSS), prognostic nomogram, risk factors, American joint committee on cancer staging (AJCC), surveillance, epidemiology, end results database (SEER database)

## Abstract

**Background and aims:**

Adenocarcinoma is one of the most common pathological types of gastric cancer. The aims of this study were to develop and validate prognostic nomograms that could predict the probability of cancer-specific survival (CSS) for gastric adenocarcinoma (GAC) patients at 1, 3, and 5 years.

**Methods:**

In total, 7747 patients with GAC diagnosed between 2010 and 2015, and 4591 patients diagnosed between 2004 and 2009 from the Surveillance, Epidemiology, and End Results (SEER) database were included in this study. The 7747 patients were used as a prognostic cohort to explore GAC-related prognostic risk factors. Moreover, the 4591 patients were used for external validation. The prognostic cohort was also divided into a training and internal validation sets for construction and internal validation of the nomogram. CSS predictors were screened using least absolute shrinkage and selection operator regression analysis. A prognostic model was built using Cox hazard regression analysis and provided as static and dynamic network-based nomograms.

**Results:**

The primary site, tumor grade, surgery of the primary site, T stage, N stage, and M stage were determined to be independent prognostic factors for CSS and were subsequently included in construction of the nomogram. CSS was accurately estimated using the nomogram at 1, 3, and 5 years. The areas under the curve (AUCs) for the training group at 1, 3, and 5 years were 0.816, 0.853, and 0.863, respectively. Following internal validation, these values were 0.817, 0.851, and 0.861. Further, the AUC of the nomogram was much greater than that of American Joint Committee on Cancer (AJCC) or SEER staging. Moreover, the anticipated and actual CSS values were in good agreement based on decision curves and time-calibrated plots. Then, patients from the two subgroups were divided into high- and low-risk groups based on this nomogram. The survival rate of high-risk patients was considerably lower than that of low-risk patients, according to Kaplan–Meier (K-M) curves (*p*<0.0001).

**Conclusions:**

A reliable and convenient nomogram in the form of a static nomogram or an online calculator was constructed and validated to assist physicians in quantifying the probability of CSS in GAC patients.

## 1 Introduction

Gastric cancer is the fifth most prevalent cancer that is diagnosed and the second leading cause of cancer mortality globally (1). Gastric adenocarcinoma (GAC) is the most common type of gastric cancer pathologically. Global gastric cancer-related incidence and mortality rates have declined, but they have still increased in some regions (2, 3).

Complete tumor resection with standardized D2 lymphadenectomy remains the main treatment for gastric cancer, whereas endoscopy and chemotherapy are the main treatment measures for early and advanced gastric cancer (4). TNM staging is still the clinical basis of gastric cancer treatment and prognostics based on the current NCCN guidelines (5). However, traditional TNM staging also has its own limitations (6, 7).

The prognosis of gastric cancer often varies among individuals because of different genotypes and molecular genetic characteristics, which could also have an important role in prognosis and treatment strategies (8). Different pathological types of gastric cancer have different prognoses. Therefore, there is a need to develop a personalized predictive model for the prognosis of GAC and to optimize treatment strategies. Predictive models have become widely accepted as reliable tools to help assess the prognosis and in clinical decision-making for numerous malignancies, including cervical cancer, esophageal cancer, and liver cancer (9–11). This study explored prognostic factors that could predict cancer-specific death in patients with GAC based on the Surveillance, Epidemiology and End Results (SEER) database and constructed and validated a practical prognostic model for optimizing clinical management and individualized patient counseling.

## 2 Materials and methods

### 2.1 Study design and data extraction

The data were retrieved using SEER*Stat 8.4.0 software (www.seer.cancer.gov) and analyzed retrospectively. The International Classification of Diseases for Oncology, Third Revision (ICD-O-3) was used to identify cases of malignant adenocarcinoma. CS16.0–16.6 comprised the primary tumor location codes, and 8140/3, 8144/3, 8210/3, 8211/3, 8255/3, 8260/3, 8261/3, 8262/3, 8263/3, 8310/3, 8323/3, 8480/3, 8481/3, and 8490/3 were the histological codes.

Histologically confirmed malignant adenocarcinoma with its primary site being the stomach, a diagnosis between 2010 and 2015, and known survival months and reason for death were the inclusion criteria. Exclusion criteria included other histological types of gastric cancer, such as undifferentiated carcinoma, neuroendocrine tumor, adenosquamous carcinoma, mesenchymal tumor, or mixed epithelial and mesenchymal tumor as adenosarcoma; diagnosis by autopsy or death certificate; unknown cause of death; and unknown duration of follow-up or survival.

Because the SEER database is a public open database, no ethical review board permission is necessary. The SEER program offers signed authorizations and licenses for accessing and utilizing the datasets. Patients diagnosed between 2010 and 2015 (n=7,747) were utilized to investigate the prognostic characteristics associated with cancer-specific survival (CSS). Meanwhile, all patients were separated into training and internal validation groups at a 7:3 ratio. The following data were extracted from the SEER database: age, race, sex, year of diagnosis, histologic subtype, grade, primary site, SEER summary stage (local, regional, or distant), and American Joint Committee on Cancer (AJCC) stage, tumor, lymph node, metastasis (TNM) stage, AJCC stage, primary site surgery, radiation therapy, chemotherapy, tumor size, number of regional lymph nodes (LNs) examined, number of positive LNs, number of cases of multiple primary tumors, survival time, cause of death, and vital status.

### 2.2 Outcomes and predictors

The primary result of this study was mortality caused by GAC. The survival time was measured from the date of diagnosis to the date of death or the final follow-up consultation. Participants with uncertain causes of death, unknown survival times, or zero survival times were omitted from the research. T stage, N stage, M stage, AJCC stage, primary site, primary site surgery (yes or no), radiation (yes or no), chemotherapy (yes or no), tumor size, number of regional LNs investigated, number of positive LNs, tumor size, and number of multiple main tumors. These factors were included as candidate predictors in the follow-up analysis. Finally, 13 variables with non-zero coefficients, associated values, and the likelihood of deviation were discovered. With a 1 standard error criteria, these variables were included.

### 2.3 Transformation of continuous variables

Nonlinear associations between continuous variables and outcomes were detected by performing restricted cubic spline analysis. As a result, they were transformed to categorical variables. Age was categorized as follows: ≤60 and >60 years depending on the optimal cutoff value. Tumor size was stratified into three groups according to previous studies as follows: <20, 20–30, and >30 mm (12, 13). Referring to earlier research, the number of LNs investigated was divided into three categories, specifically 0, 1–15, and >=16, and the number of positive LNs was divided into three groups, 0, 1–3, and >3 (5, 14, 15). Owing to the rarity of grades III and IV tumors, cases with these grades were combined into a single category. As a result, the tumor grade was divided into two groups, grade I plus II and grade III plus IV. The three SEER summary stages were localized, regional, and distant. The stages of the AJCC were renamed I, II, III, and IV. The terms T1, T2, T3, and T4; N0 and N1; and M0 and M1, respectively, were used to denote the AJCC T, N, and M stages. There were two categories created for the number of primary tumors, one primary group and more than or equal to two primary groups.

### 2.4 Predictor selection

All variables were included in a least absolute shrinkage and selection operator (LASSO) regression analysis using the development cohort to discover possible risk factors for cancer-specific mortality (CSD). Here, as the penalty increases, the estimates of the weaker components fall toward zero and can therefore be used to filter variables. The predictive efficacy of models with 5–10 variables was examined to refine the model for clinical use. When 7–10 variables were added to the model, the area under the receiver operating characteristic (ROC) curve (AUC) was always greater than 0.800. As a result, seven factors with corresponding values were discovered for further analyses, including grade, T stage, N stage, M stage, primary site, number of positive LNs, and operation at the primary site. In a multiple covariance analysis of the seven variables, the variance inflation factors (VIFs) for the number of positive N stages and LNs were 4.2736 and 4.6644, respectively, whereas the VIF for the other five variables were less than 2 (data not shown). Multicollinearity between variables reduces the stability and accuracy of the model. Therefore, the number of positive LNs was excluded. Six variables, including grade, T stage, N stage, M stage, primary site, and surgery at the primary site, were used for model construction.

### 2.5 Construction and validation of prognostic nomogram

A prognostic nomogram based on Cox proportional risk regression analysis was established. The training group was used for nomogram construction, and the testing group was utilized for validation. The nomogram could intuitively predict the probability of CSS at 1, 3, and 5 years. ROC curves and calibration curves were plotted for the 1-, 3-, and 5-year CSS. We created a free online web calculator based on the prognostic nomogram to allow doctors to calculate CSS with 95% confidence intervals (CIs). The time-dependent ROC of the nomogram was also compared to the time-dependent ROC of AJCC staging and SEER composite staging, with a larger AUC value suggesting superior prognostic prediction accuracy. A Kaplan–Meier (K–M) survival curve according to the risk score of the prognostic nomogram was also plotted for the training and testing groups. Finally, 4591 patients diagnosed between 2004 and 2009 were used for external validation.

### 2.6 Statistical analysis

All statistical analyses were carried out using R (version 4.1.2) and SPSS 19.0. Categorical variables are presented as numbers and percentages. CSS potential predictors were chosen using LASSO regression analysis and multivariate Cox regression analysis. The prognostic nomogram was created using Cox hazard regression analysis and was presented as a static nomogram and a dynamic network-based nomogram. Calibration, decision curve analysis, and time-dependent ROC curves were also plotted to evaluate the nomogram. Finally, risk scores for each patient in the training and test groups were calculated using the nomogram. Patients in each group were divided into high and low risk groups based on the median risk score. K–M curves and a log-rank test were further used to assess survival differences between high- and low-risk groups. The R packages glmnet, caret, mctest, dcurves, pROC, regplot, rms, survival, timeROC, survminer, and DynNom were used for the analysis. A *p*-value <0.05 was considered statistically significant.

The p-values in **Tables 1**, **2** were obtained from the CreateTableOne function in the Tableone package in R software by Chi-square test. Prognostic models were constructed based on independent predictors using the survivor package in R software, the regplot package to construct the nomogram, and the DynNom package to further construct the dynamic nomogram. pROC package was used to plot ROC curves and its AUC was used to assess the discriminatory power of the nomogram. In addition, the timeROC package was used to generate ROC curves over time and to compare the AUC of different stages with the nomogram. The calibration curves were plotted using the survival package and the rms package, and the dcurves package was used to plot DCA. Finally, all patients were divided into high-risk and low-risk groups based on the median risk score predicted by the nomogram, and the survival curves were validated for the prognostic value of the nomogram using the log-rank test, using the survival package and the survminer package to plot K-M curves for visualization.

**Table 1:** Baseline characteristics of GAC patients.

Characteristics	Training group(*n*=5423)	Validation group (*n*=2324)	*p*
Age, years			0.196
≤60	1679 (31.00%)	685 (29.48%)	
>60	3744 (69.00%)	1639 (70.52%)	
Race			0.233
Black	722 (13.30%)	312 (13.43%)	
Other	1137 (21.00%)	526 (22.63%)	
White	3564 (65.70%)	1486 (63.94%)	
Sex			0.041
Female	2025 (37.30%)	925 (39.80%)	
Male	3398 (62.70%)	1399 (60.20%)	
Primary Site^*^			0.342
C16.0	1588 (29.30%)	645 (27.75%)	
C16.1	234 (4.30%)	82 (3.53%)	
C16.2	694 (12.80%)	314 (13.51%)	
C16.3	1651 (30.40%)	715 (30.77%)	
C16.4	223 (4.10%)	111 (4.78%)	
C16.5	720 (13.30%)	312 (13.43%)	
C16.6	313 (5.80%)	145 (6.24%)	
Grade			0.117
I–II	2026 (37.40%)	824 (35.46%)	
III–IV	3397 (62.60%)	1500 (64.54%)	
SEER Stage			0.845
Distant	1042 (19.20%)	434 (18.67%)	
Localized	1816 (33.50%)	779 (33.52%)	
Regional	2565 (47.30%)	1111 (47.81%)	
AJCC Stage			0.974
I	1560 (28.80%)	671 (28.87%)	
II	1250 (23.00%)	526 (22.63%)	
III	1714 (31.60%)	744 (32.01%)	
IV	899 (16.60%)	383 (16.48%)	
AJCC T stage			0.690
T1	1595 (29.40%)	658 (28.31%)	
T2	686 (12.60%)	312 (13.43%)	
T3	1945 (35.90%)	837 (36.02%)	
T4	1197 (22.10%)	517 (22.25%)	
AJCC N stage			0.691
N0	2448 (45.10%)	1034 (44.49%)	
N1	1379 (25.40%)	603 (25.95%)	
N2	803 (14.80%)	329 (14.16%)	
N3	793 (14.60%)	358 (15.40%)	
AJCC M stage			0.942
M0	4524(83.40%)	1941(83.52%)	
M1	899(16.60%)	383(16.48%)	
Surg Prim Site			0.585
No	1112 (20.50%)	490 (21.08%)	
Yes	4311 (79.50%)	1834 (78.92%)	
Radiation			0.954
No	3726 (68.70%)	1599 (68.80%)	
Yes	1697 (31.30%)	725 (31.20%)	
Chemotherapy			0.455
No	2359 (43.50%)	1033 (44.45%)	
Yes	3064 (56.50%)	1291 (55.55%)	
LNs examined			0.990
≥16	2182 (40.20%)	939 (40.40%)	
1~15	1838 (33.90%)	786 (33.82%)	
None	1403 (25.90%)	599 (25.77%)	
LNs positive			0.639
>3	1200 (22.10%)	537 (23.11%)	
1~3	946 (17.40%)	401 (17.25%)	
None	3277 (60.40%)	1386 (59.64%)	
Tumor size, mm			0.064
<20	1000 (18.40%)	390 (16.78%)	
>30	3299 (60.80%)	1478 (63.60%)	
20-30	1124 (20.70%)	456 (19.62%)	
Multi-primary tumors			0.474
≥2	1195 (22.00%)	530 (22.81%)	
1	4228 (78.00%)	1794 (77.19%)	

Primary Site^*^: C16.0-cardia, NOS; C16.1-fundus of stomach; C16.2-body of stomach; C16.3-gastric antrum; C16.4-pylorus; C16.5-lesser curvature of stomach NOS; C16.6-greater curvature of stomach NOS; LN, lymph node; AJCC, American Joint Committee on Cancer; SEER, Surveillance, Epidemiology, and End Results.

**Table 2:** Baselines of prognostic cohort and external cohort.

Characteristics	Prognostic cohort(n=7747)	External cohort(n=4591)	*p*
Age, years (%)			0.213
<=60	2364 (30.5)	1451 (31.6)	
>60	5383 (69.5)	3140 (68.4)	
Sex (%)			0.239
Female	2950 (38.1)	1798 (39.2)	
Male	4797 (61.9)	2793 (60.8)	
Primary Site* (%)			<0.001
C16.0	2233 (28.8)	1099 (23.9)	
C16.1	316 (4.1)	219 (4.8)	
C16.2	1008 (13.0)	554 (12.1)	
C16.3	2366 (30.5)	1524 (33.2)	
C16.4	334 (4.3)	237 (5.2)	
C16.5	1032 (13.3)	656 (14.3)	
C16.6	458 (5.9)	302 (6.6)	
Grade (%)			<0.001
I~II	2850 (36.8)	1414 (30.8)	
III~IV	4897 (63.2)	3177 (69.2)	
T (%)			<0.001
T1	2253 (29.1)	1230 (26.8)	
T2	998 (12.9)	1758 (38.3)	
T3	2782 (35.9)	944 (20.6)	
T4	1714 (22.1)	659 (14.4)	
N (%)			<0.001
N0	3482 (44.9)	1919 (41.8)	
N1	1982 (25.6)	1752 (38.2)	
N2	1132 (14.6)	662 (14.4)	
N3	1151 (14.9)	258 (5.6)	
M (%)			<0.001
M0	6465 (83.5)	3568 (77.7)	
M1	1282 (16.5)	1023 (22.3)	
SEER.Stage (%)			<0.001
Distant	1476 (19.1)	1087 (23.7)	
Localized	2595 (33.5)	1250 (27.2)	
Regional	3676 (47.5)	2254 (49.1)	
Surg.Prim.Site (%)			0.001
No	1602 (20.7)	1072 (23.4)	
Yes	6145 (79.3)	3519 (76.6)	

Primary Site^*^: C16.0-cardia, NOS; C16.1-fundus of stomach; C16.2-body of stomach; C16.3-gastric antrum; C16.4-pylorus; C16.5-lesser curvature of stomach NOS; C16.6-greater curvature of stomach NOS.

## 3 Results

### 3.1 Demographic and clinical characteristics

This study included 7747 people diagnosed with GAC in the SEER database between 2010 and 2015. The training group (n=5423) was used to construct the nomogram, and the testing group was used for internal validation. The baseline characteristics of all patients are shown in **Table 1**.

### 3.2 Predictor selection

**Figure 1:**
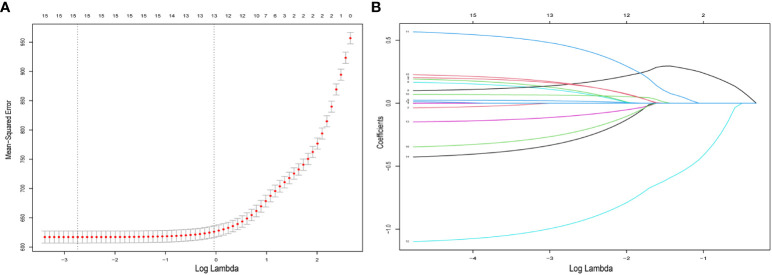
Predictor selection for LASSO regression analysis. **(A)** The mean−squared error was plotted versus log lambda. The left vertical dotted line shows the optimal values with the fewest criteria, whereas the right vertical dotted line reflects the single standard error criterion. **(B)** LASSO coefficient profiles of the 15 variables. LASSO, least absolute shrinkage and selection operator.

All patients comprised a cohort to explore the relevant prognostic factors. To prevent variable overfitting and simplify the model, LASSO regression analysis was employed to penalize the absolute values of the coefficients (**Figure 1**). Six variables, including grade, T stage, N stage, M stage, primary site, and primary site of surgery, were identified by combining the findings of LASSO and multivariate cox analyses. We also performed univariate and multivariate cox analyses for comparison (**Table 3**). Six candidate variables were included in multivariate cox regression analyses, all as independent risk factors for CSS.

**Table 3:** The results of Univariate and multivariate Cox analysis.

Characteristics	Univariate Cox analysis			Multivariate Cox analysis		
	HR	*P*	95%CI	HR	*P*	95%CI
Grade						
I~II	Reference			Reference		
III~IV	1.826	0.000	1.703-1.959	1.273	0.000	1.185-1.369
T						
T1	Reference			Reference		
T2	1.256	0.000	1.107-1.425	1.117	0.091	0.982-1.270
T3	2.147	0.000	1.961-2.351	1.624	0.000	1.471-1.793
T4	4.140	0.000	3.772-4.544	2.335	0.000	2.101-2.594
N						
N0	Reference			Reference		
N1	2.114	0.000	1.947-2.294	1.275	0.000	1.167-1.393
N2	2.237	0.000	2.036-2.458	1.686	0.000	1.523-1.866
N3	3.654	0.000	3.349-3.987	2.643	0.000	2.393-2.920
M						
M0	Reference			Reference		
M1	4.827	0.000	4.498-5.18	1.970	0.000	1.814-2.139
Primary Site*						
C16.0	Reference			Reference		
C16.1	1.776	0.000	1.522-2.072	1.637	0.000	1.401-1.912
C16.2	1.368	0.000	1.230-1.522	1.452	0.000	1.301-1.620
C16.3	1.371	0.000	1.260-1.492	1.593	0.000	1.456-1.742
C16.4	1.444	0.000	1.233-1.692	1.705	0.000	1.451-2.005
C16.5	1.395	0.000	1.257-1.549	1.407	0.000	1.264-1.567
C16.6	1.565	0.000	1.365-1.796	1.688	0.000	1.468-1.940
Surg Prim Site						
No	Reference			Reference		
Yes	0.230	0.000	0.215-0.246	0.231	0.000	0.213-0.251

Primary Site^*^: C16.0-cardia, NOS; C16.1-fundus of stomach; C16.2-body of stomach; C16.3-gastric antrum; C16.4-pylorus; C16.5-lesser curvature of stomach NOS; C16.6-greater curvature of stomach NOS.

### 3.3 Construction of the prognostic nomogram

**Figure 2:**
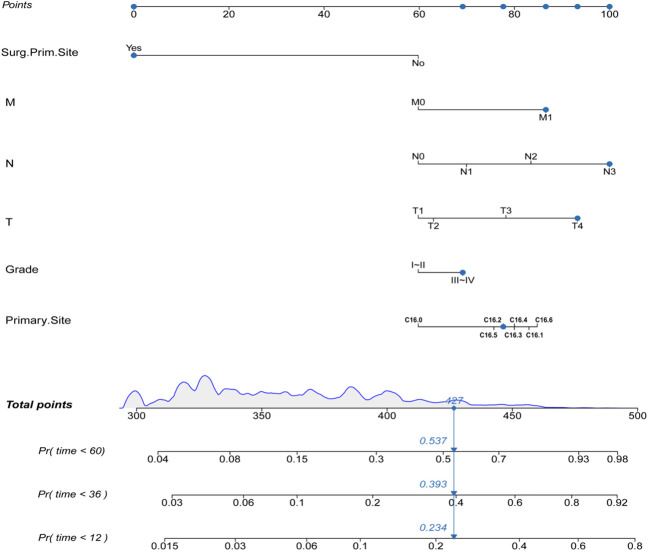
The prognostic nomogram for GAD patients.

**Figure 3:**
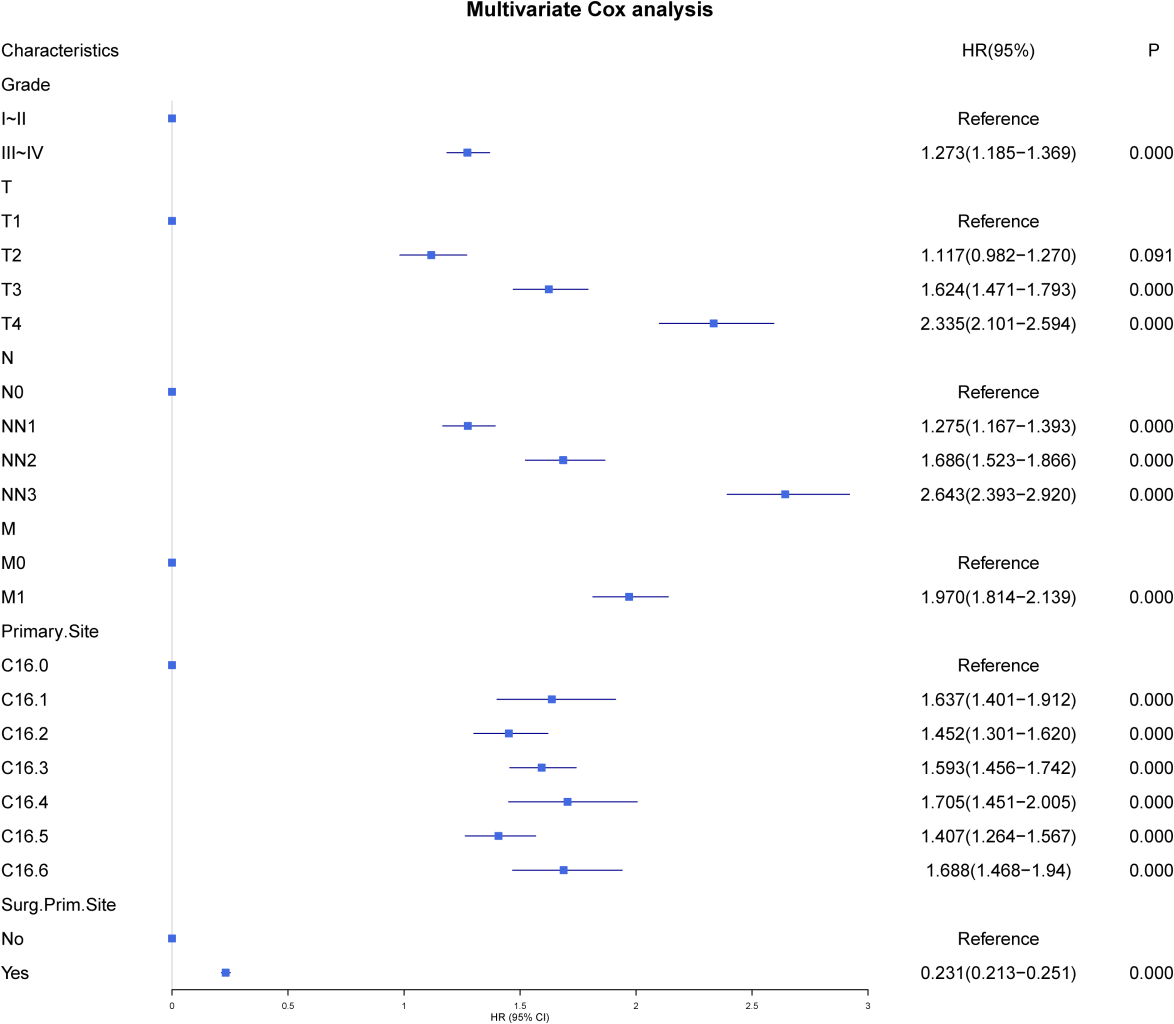
Forest plot of multivariate cox analysis.

Construction of the prognostic nomogram using the tumor grade, T-stage, N-stage, M-stage, primary site, and primary site of surgery in the training group is shown in **Figure 2**. A testing group was further used for nomogram validation. The results of the multivariate cox analyses were summarized as a forest plot showing the independent effect of predictors of CSS on GAC patients using hazard ratios (HRs) and the 95% CI (**Figure 3**). Surgery at the primary site was a favorable factor for prognosis (HR=0.269, 95% CI: 0.248–0.292, *p*<0.001). The remaining five prognostic factors were all strongly associated with poorer prognosis. Moderately differentiated and poorly or undifferentiated tumors were associated with a relatively poor prognosis compared to that with well differentiated tumors (HR=1.297, 95% CI: 1.207−1.394, *p*<0.001). Compared to that with T1 stage disease, the prognosis of T2 stage gastric cancer was not significantly different, but T3 and T4 stages were associated with a poorer prognosis (HR=1.117, 95% CI: 0.982−1.270, *p* = 0.091; HR=1.624, 95% CI: 1.471−1.793, *p*<0.001; HR=2.335, 95% CI: 2.101−2.920, *p*<0.001, respectively). Distant metastasis (HR=1.970, 95% CI: 1.814−2.139, *p*<0.001) was associated with a poor prognosis. Compared to that with the cardia site, all other sites were risk factors for gastric cancer prognosis (C16.1: HR=1.776, 95%CI: 1.522–2.072, *p*<0.001; C16.2: HR=1.368, 95%CI: 1.23–1.522, *p*<0.001; C16.3: HR=1.371, 95%CI: 1.26–1.492, *p*<0.001; C16.4: HR=1.444, 95%CI: 1.233–1.692, *p*<0.001; C16.5: HR=1.395, 95%CI: 1.257–1.549, *p*<0.001; C16.6: HR=1.565, 95%CI: 1.365–1.796, *p*<0.001).

### 3.4 Internal validation of the nomogram

**Figure 4:**
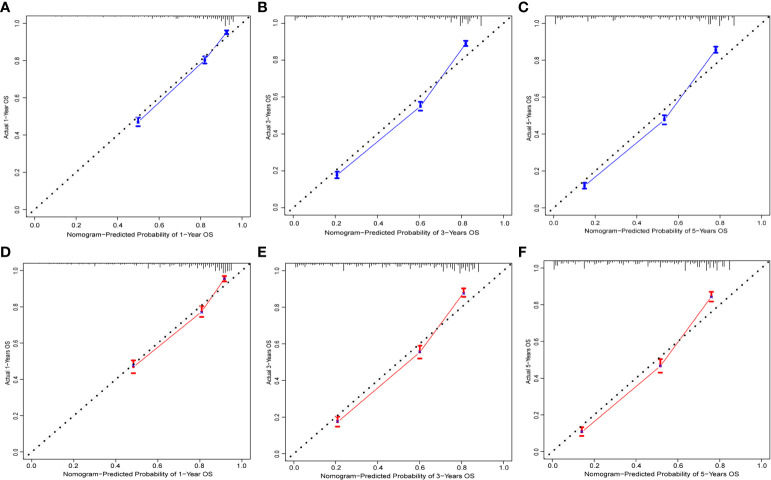
Calibration curves for nomogram. The 1-**(A)**, 3-**(B)**, and 5-year **(C)** calibration curves for nomogram in the training group. The 1-**(D)**, 3-**(E)**, and 5-year **(F)** calibration curves for nomogram in the validation group.

**Figure 5:**
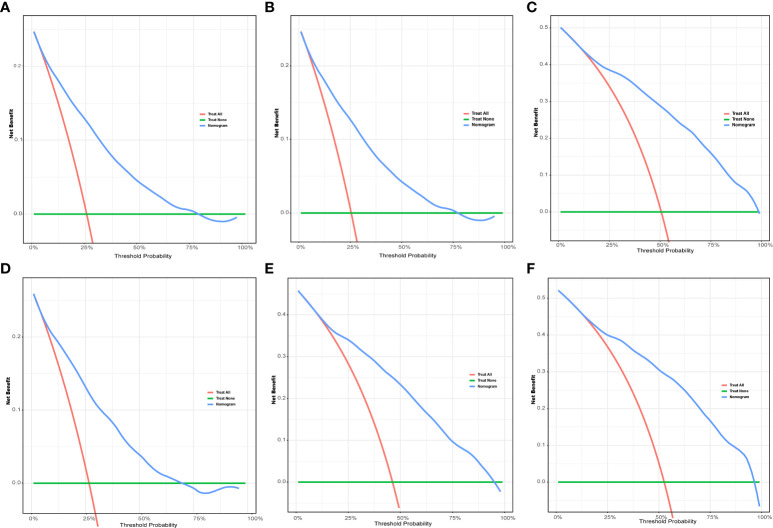
The DCA for nomogram. The 1-**(A)**, 3-**(B)**, and 5-year **(C)** DCA for nomogram in the training group. The 1-**(D)**, 3-**(E)**, and 5-year **(F)** DCA for nomogram in the validation group. DCA, Decision analysis curve.

**Figure 6:**
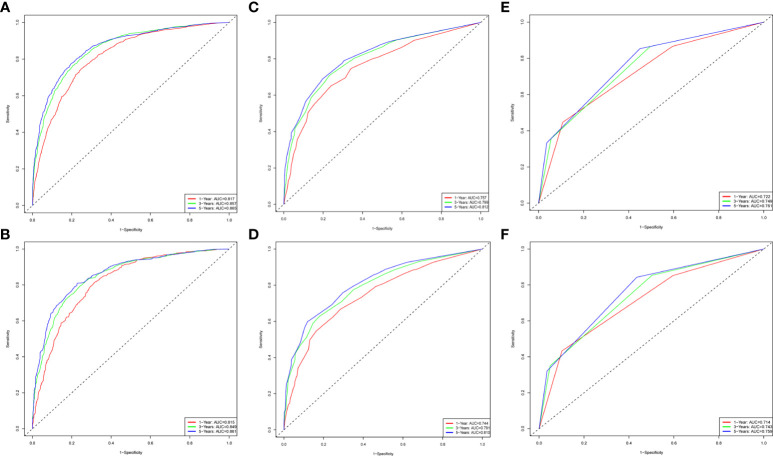
The time-dependent ROC curves for nomogram, SEER stage and AJCC stage. The time-dependent ROC curves 1-,3-and 5-year for nomogram in the training group **(A)** and validation group **(B)**. The time-dependent ROC curves 1-,3-and 5-year for SEER stage in the training group **(C)** and validation group **(D)**. The time-dependent ROC curves 1-,3-and 5-year for the AJCC stage in the training group **(E)** and validation group **(F)**.

To evaluate the performance of the nomogram, we performed internal validation of the nomogram and plotted calibration curves, DCA, and time-dependent ROC curves. The calibration curves for both the training group (**Figures 4A-C**) and testing group (**Figures 4D-F**) showed that the nomogram has good calibration capability. The DCA of the training (**Figures 5A-C**) and testing groups (**Figures 5D-F**) showed that the nomogram has good clinical applicability. Meanwhile, the time-dependent ROCs for the nomogram of the two groups were also plotted. The 1-, 3-, and 5-year AUCs of the nomogram for the training group were 0.817, 0.857, and 0.865, respectively (**Figures 6A**). The AUCs of the nomogram in the testing group were 0.815, 0.845, and 0.861, respectively (**Figures 6B**). The time-dependent ROCs of the AJCC stage (**Figures 6C, D**) and SEER stage (**Figures 6E, F**) in the training and testing groups were also plotted. The AUC of the nomogram was significantly better than that of the AJCC stage and SEER stage. Based on these results, the model has reliable discriminatory capability.

**Figure 7:**
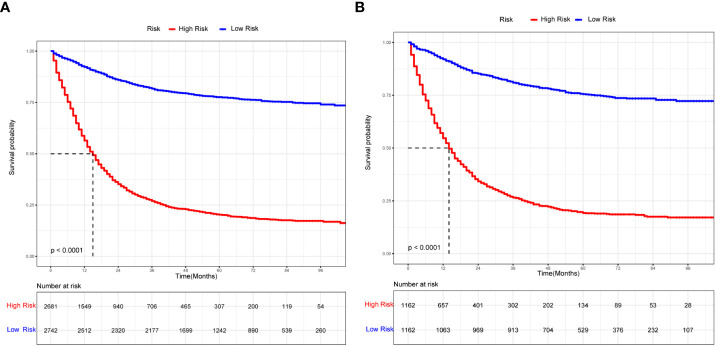
The K-M curves for nomogram. **(A)** The K-M curve for nomogram in the training group. **(B)** The K-M curve for nomogram in the validation group.

**Figure 8:**
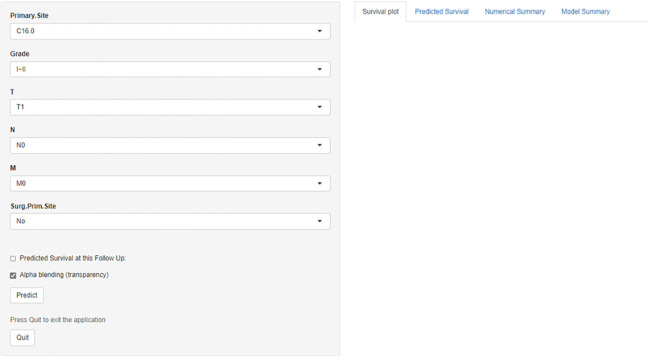
The interface for dynamic nomogram.

Prognosis was separated into high- and low-risk groups based on the median value of the prognostic score generated by the prognostic nomogram to further examine the viability and validity of the prediction model. The K–M survival curves showed substantially different prognoses (*p*<0.001), demonstrating that the model can identify individuals at high risk of cancer-specific mortality (**Figure 7**). The K–M survival curves revealed that high-risk patients had considerably poorer CSS than low-risk individuals. Finally, we further generated an online dynamic nomogram to facilitate the clinical application of the nomogram, and **Figure 8** illustrates the operational interface of the dynamic nomogram.

### 3.5 External validation of the nomogram

**Figure 9:**
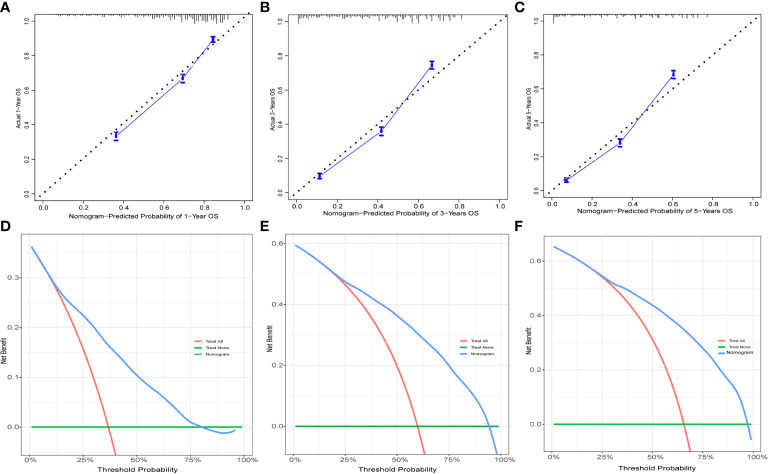
The calibration curves and DCA for external validation. The 1-**(A)**, 3-**(B)**, and 5-year **(C)** calibration curves for nomogram in the external validation group. The 1-**(D)**, 3-**(E)**, and 5-year **(F)** DCA for nomogram in the external validation group.

**Figure 10:**
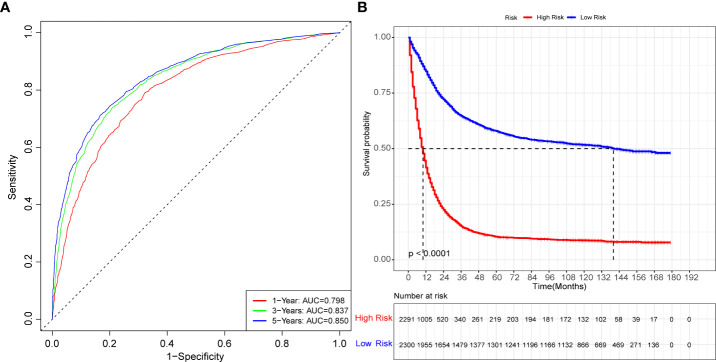
The time-dependent ROC curves and K-M curves for external validation. The time-dependent ROC curves 1-,3-and 5-year for nomogram in the external validation group **(A)**. The K-M curve for nomogram in the external validation group **(B)**.

The **Table 2** shows the baseline of important variables between the two cohorts. In the external validation, the ROC (**Figures 9A-C**) and DCA (**Figures 9D-F**) shown that the nomogram robust calibration capabilities and clinical value. The AUCs of 1-, 3-, and 5-year time-dependent ROC for nomogram were 0.798, 0.837, and 0.850 (**Figure 10A**). Meanwhile, the K-M curves indicate statistically significant differences in CSS between high and low risk group (**Figure 10B**).

## 4 Discussion

The discovery of independent prognostic markers and the creation of reliable prediction models are critical for counseling, better treatment planning, and follow-up for patients with GAC. This study used SEER data to identify the tumor grade, T stage, primary site, N stage, M stage, and surgery at the primary site as independent prognostic variables for GAC and created a new nomogram to predict 1-, 3-, and 5-year survival. The prediction nomogram performed well and had good discriminating power. Furthermore, the calibration curves, DCA, and time-dependent ROC curve validation results revealed that the model has great discriminative capacity and good agreement between anticipated and actual observed outcomes. Overall, the results of this study show that the prognostic nomogram has high predictive performance based on both training and testing groups. Each prognostic feature was measured and presented *via* a static nomogram, allowing the 1-, 3-, and 5-year GAC-specific survival probabilities to be predicted individually. For easier application of the model, we created a calculator based on a network of the static nomogram (https://glfl993823.shinyapps.io/GAC_CSS/), which allows for the likelihood of survival, with the 95% CI, to be calculated by entering the values of the six variables. Clinicians will be able to use our prediction model to assess individual risk based on a variety of known characteristics and to acutely individualize therapy and follow-up for GAC patients. Because individuals classed as high risk are more likely to succumb to CSD, they should be treated completely and constantly monitored. The prognostic nomogram contributes predictive value when combined with primary site surgery, the primary site, and tumor grade when compared to that of the AJCC and SEER staging systems, indicating that it can have a complementary role.

Although this study was not the first to explore a GAC-related prognostic nomogram, its predictive performance was significantly better than that of other predictive models. Shi et al. (16) constructed a prognostic nomogram for gastric cancer based on 11 variables, including sex, age, marital status, race, SEER stage, grade, T stage, N stage, M stage, tumor size, and surgery, with the AUCs for CSS being 0.700 and 0.706 in the training and validation group, respectively. Yu et al. (17) established a prognostic nomogram based on the tumor size, SEER stage, and primary site for young gastric cancer patients, with the AUCs for 3- and 5- year CSS being approximately 0.725–0.773 in both the training and testing groups. With the development of medical technology, the application of molecular diagnosis in malignant tumors is becoming increasingly widespread. In addition, an increasing number of molecular mechanisms and sequencing technologies are being used to facilitate the study of tumors (18–20). Related molecular mechanisms and sequencing technologies are expected to play an important role in the prognosis of cancer patients. The immune infiltration score (21), DNA methylation (22), and LncRNAs (23, 24) are all closely related to the prognosis of gastric cancer. Although research at the molecular level can provide an individualized diagnosis and treatment basis for gastric cancer patients, it is still difficult to implement tests at the molecular level in clinical practice.

Many studies have also investigated the prognostic factors associated with gastric cancer. It has been shown that patients with gastric cancer in the greater curvature have a longer overall survival than those with the sinus, gastroesophageal junction, and lesser curvature as the primary sites (25). In addition, cardia cancer is positively associated with bone metastasis, among gastric cancers, which is one of its independent risk factors (26). This primary site was also found to be an independent prognostic factor for GAC in this study. Moreover, tumor size, tumor stage, and tumor grade are all prognostic factors for gastric cancer patients (27). Surgery is also an important prognostic factor for gastric cancer patients (28). The following characteristics were found to be independent predictive factors in this study and were incorporated in the model: tumor grade, stage T, stage N, stage M, primary location, and surgery at primary site.

In this study, a new prognostic nomogram for GAC patients was developed based on the SEER database, and the internal and external validation results showed that the model provides a more personalized assessment of GAC prognosis than the AJCC staging system. However, the present study has several limitations. First, the model is based on a retrospective analysis and inevitably suffers from selection bias. Second, the SEER database does not contain information on some potential independent prognostic variables, such as laboratory tests and imaging data, and these indicators could further affect the predictive performance of the model. Finally, the model still requires validation with external data, especially in Asian medical institutions, to increase applicability of the model.

Moreover, the small percentage of patients with gastric cancer with metastasis in this study, and the presence of some patients with conversion surgery in the SEER database cannot be excluded. The presence of these factors may have a potential impact. It has been shown that palliative surgery at the primary site does not prolong survival in patients with metastatic gastric cancer (29). Related studies also suggest that surgery may be a feasible option in liver metastases in gastric cancer (30), but further studies with large sample data are still needed.

## 5 Conclusion

Ultimately, this study identified the tumor grade, T stage, N stage, M stage, tumor primary site, and surgery at primary site as independent prognostic factors and constructed a new nomogram to predict 1-, 3-, and 5-year CSS for patients with GAC. Nomogram showed good discriminatory power and clinical applicability. The static nomogram or online prediction tool will assist clinicians in quantifying the risk of death and calculating the probability of death to develop individualized treatment and follow-up strategies. However, further validation of this prediction model is needed based on large prospective multicenter studies.

## Data availability statement

Publicly available datasets were analyzed in this study. This data can be found here: https://seer.cancer.gov/.

## Ethics statement

Ethical review and approval was not required for the study on human participants in accordance with the local legislation and institutional requirements. Written informed consent for participation was not required for this study in accordance with the national legislation and the institutional requirements.

## Author contributions

GN and HLZ conceived and designed the study. DX, HJZ, and JY performed a literature search. GN and HLZ wrote the manuscript. XL critically reviewed the manuscript. All authors contributed to the article and approved the submitted version.
